# Identification and functional characterization of *de novo* variant in the *SYNGAP1* gene causing intellectual disability

**DOI:** 10.3389/fgene.2023.1270175

**Published:** 2023-10-19

**Authors:** Boxuan Li, Yu Wang, Dong Hou, Zhen Song, Lihua Zhang, Na Li, Ruifang Yang, Ping Sun

**Affiliations:** ^1^ Center of Prenatal Diagnosis, Department of Obstetrics and Gynecology, Qilu Hospital of Shandong University, Jinan, China; ^2^ Center for Reproductive Medicine, Department of Obstetrics and Gynecology, Qilu Hospital of Shandong University, Jinan, China; ^3^ Suzhou Research Institute of Shandong University, Suzhou, China

**Keywords:** SYNGAP1, whole exome sequencing (WES), intellectual disability, minigene, variant

## Abstract

**Background:** Intellectual disability (ID) is defined by cognitive and social adaptation defects. Variants in the *SYNGAP1* gene, which encodes the brain-specific cytoplasmic protein SYNGAP1, are commonly associated with ID. The aim of this study was to identify novel *SYNGAP1* gene variants in Chinese individuals with ID and evaluate the pathogenicity of the detected variants.

**Methods:** Whole exome sequencing (WES) was performed on 113 patients diagnosed with ID. In the study, two *de novo* variants in *SYNGAP1* were identified. Sanger sequencing was used to confirm these variants. Minigene assays were used to verify whether the *de novo* intronic variant in *SYNGAP1* influenced the normal splicing of mRNA.

**Results:** Two *de novo* heterozygous pathogenic variants in *SYNGAP1,* c.333del and c.664-2A>G, were identified in two ID patients separately. The c.333del variant has been reported previously as a *de novo* finding in a child with ID, while the c.664-2A>G variant was novel *de novo* intronic variant, which has not been reported in the literature. Functional studies showed that c.664-2A>G could cause aberrant splicing, resulting in exon 7 skipping and a 16bp deletion within exon 7.

**Conclusion:** We identified two *de novo* pathogenic heterozygous variants in *SYNGAP1* in two patients with ID, among which the c.664-2A>G variant was a novel *de novo* pathogenic variant. Our findings further enrich the variant spectrum of the *SYNGAP1* gene and provide a research basis for the genetic diagnosis of ID.

## Introduction

Intellectual disability (ID) is characterized by cognitive and social adaptation defects, which typically occur before the age of 18 (Chelly et al., 2006). ID is the most common severe disability in children, affecting approximately 1%–3% of the population (Goldenberg and Saugier-Veber, 2010). ID is classified as either a syndromic or non-syndromic (NSID) form. The majority of ID patients have the NSID form of the disease, which is mainly characterized by the lack of relevant morphological, radiological, and metabolic features (Hane et al., 1996). ID patients often need lifelong rehabilitation support treatment, causing substantial psychological and economic burdens for families and society. Determining the genetic and molecular basis of ID remains a significant challenge in neuroscience.

The *SYNGAP1* gene encodes the brain-specific RAS GTPase-activating protein SYNGAP1, which is an important component of the N-methyl-D-aspartate receptor (NMDA) complex and plays a pivotal role in neuronal synaptic development, structure, and plasticity (Clement et al., 2012). *De novo* variants of *SYNGAP1* are a common cause of NSID, autism spectrum disorders (ASD), and epilepsy (Berryer et al., 2013; Mignot et al., 2016). *De novo* nonsense variants in *SYNGAP1* cause haploinsufficiency, resulting in a neurodevelopmental disorder known as intellectual developmental disorder (OMIM #612621), with phenotypes including ID, motor disorders, and epilepsy. The effects of these variants demonstrate the importance of SYNGAP1 in developing the nervous system and brain (Agarwal et al., 2019). Currently, approximately 0.7%–1% of ID cases are caused by *SYNGAP1* variants (Mignot et al., 2016).

In this study, we identified two *de novo* heterozygous pathogenic variants in the *SYNGAP1,* c. 333del and c.664-2A>G, in two patients with ID, among which the c.664-2A>G variant was a novel *de novo* pathogenic variant. The patients exhibited generalized developmental delay, motor retardation, hypotonia, and severe language impairment. To assess the impact of c.664-2A>G variant on splicing, we performed a minigene splicing assay. We found that the c.664-2A>G variant causes aberrant splicing, which would result in impaired function of the SYNGAP1 protein and consequently contribute to the occurrence of ID.

## Methods and materials

### Subjects

Two female patients from unrelated families were diagnosed with intellectual developmental disorder from a clinical cohort of 113 cases with ID from between January 2019 and January 2023 at Qilu Hospital of Shandong University. All the patients were diagnosed by experienced experts of the hospital according to the DSM-5 criteria. Among these patients with ID, 25 cases were combined with epilepsy and 49 cases were combined with other structural anomalies. The age of the children ranged from 12 months to 18 years, with a median age of 8 years. The etiology of these patients was unknown. All of these patients underwent karyotyping and chromosome microarray (CMA) analysis, and these results were inconclusive, following these samples were processed for WES. We collected peripheral blood and clinical information from their families. The families accepted the inheritance consultation and signed the informed consent form before the genetic test. This study was authorized by the Ethics Committee of Qilu Hospital of Shandong University.

### DNA extraction and whole exome sequencing (WES)

The genomic DNA for sequencing was obtained from peripheral blood. The extraction steps were conducted according to the instructions of the DNA extraction kit (Tiangen Biotech). WES was performed on the DNA from the affected individual and sequenced on NovaSeq 6000 platforms (Illumina) with 150 bp paired-end reads. Reads data were aligned with the GRCh37/hg19 human reference sequence. The single-nucleotide variants (SNVs) and other variants were called with the Genome Analysis Toolkit (GATK). The variants were annotated using Annovar software. During the annotation, several public databases such as Clinvar, gnomAD, PubMed, HGMD, dbNSFP, etc., were used. Variants with allele frequencies higher than 1% in any public databases (ExAC Browser and gnomAD) were excluded. *De novo* variants were analyzed from sequencing data by DeNovoGear software (Ramu et al., 2013). The candidate variants were confirmed in the patients with ID by Sanger sequencing.

### Minigene assay

The *SYNGAP1* c.664-2A>G variant is located at the splice-acceptor site of exon 7. We obtained the *SYNGAP1* fragment [intron6 (192bp)-Exon7 (99bp)-intron7 (547bp)] with restriction sites (KpnI and BamHI) from human genomic DNA by nested PCR amplification and then cloned it into a pcMINI plasmid using nucleic acid endonuclease and DNA ligase. The pcMINI vector contain ExonA-IntronA-multiple cloning site-IntronB-ExonB (Bioeagle Biotech Company). Exon A and Exon B simulate exon 6 and exon 8, respectively. The pcMINI-*SYNGAP1*-MUT (c.664-2A>G) plasmids were produced using a QuikChange Lightning Site-Directed Mutagenesis Kit (Agilent) with pcMINI-*SYNGAP1*-WT (wild-type) plasmid as the template. Both WT and mutant plasmids contained the whole sequence of exon 7 and a portion of the upstream and downstream intron sequences. The recombinant plasmids were transiently transfected into HEK293T and HeLa cells according to the transfection reagent instructions. After the transfected cells were cultured for 48 h, total RNA was extracted with Trizol (TaKaRa), and cDNA was acquired with HifairTM 1st Strand cDNA Synthesis SuperMix (TEASEN). The RT-PCR products was analyzed by electrophoresis on 2% agarose gels containing ethidium bromide and visualized by exposure to ultraviolet light. Each DNA band was purified by DNA Gel Exctration Kit (SIMGEN). Direct sequencing of purified RT-PCR products was performed with the Big Dye Terminator Cycler Sequencing Ready Reaction Kit (Applied Biosystems) on the ABI3730xl Genetic Analyzer (Applied Biosystems). Primers used for minigene assay of *SYNGAP1* were as follows: *SYNGAP1*-F1: 5′-AAC​TCC​TGG​GCT​CAA​GTG​AC-3′; *SYNGAP1*-R1: 5′-TGG​GTA​AAG​CTT​GGC​CAG​AT-3′; *SYNGAP1*-F2:5′-AGCACTTTGGGAGGCTGAAT-3′; *SYNGAP1*-R2: 5′-GAG​GTT​GCA​GTG​AGC​CAA​GA-3′; MINI-*SYNGAP1*-KpnI-F: 5′-GGT​AGG​TAC​CCT​GGG​GAG​GGC​CAA​AGG​ACA-3′; MINI-*SYNGAP1*-BamHI-R: 5′-TAG​TGG​ATC​CGA​GAA​TAG​CTG​ACA​GAA​CTG-3′; *SYNGAP1*-c.664-2A>G-F: 5′-TCC​ACA​CTC​CTT​TCT​GGG​TAA​CAA​CTT​CAT​C-3′; *SYNGAP1*-c.664-2A>G-R: 5′-GAT​GAA​GTT​GTT​ACC​CAG​AAA​GGA​GTG​TGG​A -3′.

## Results

### De novo heterozygous variants were identified

**FIGURE 1:**
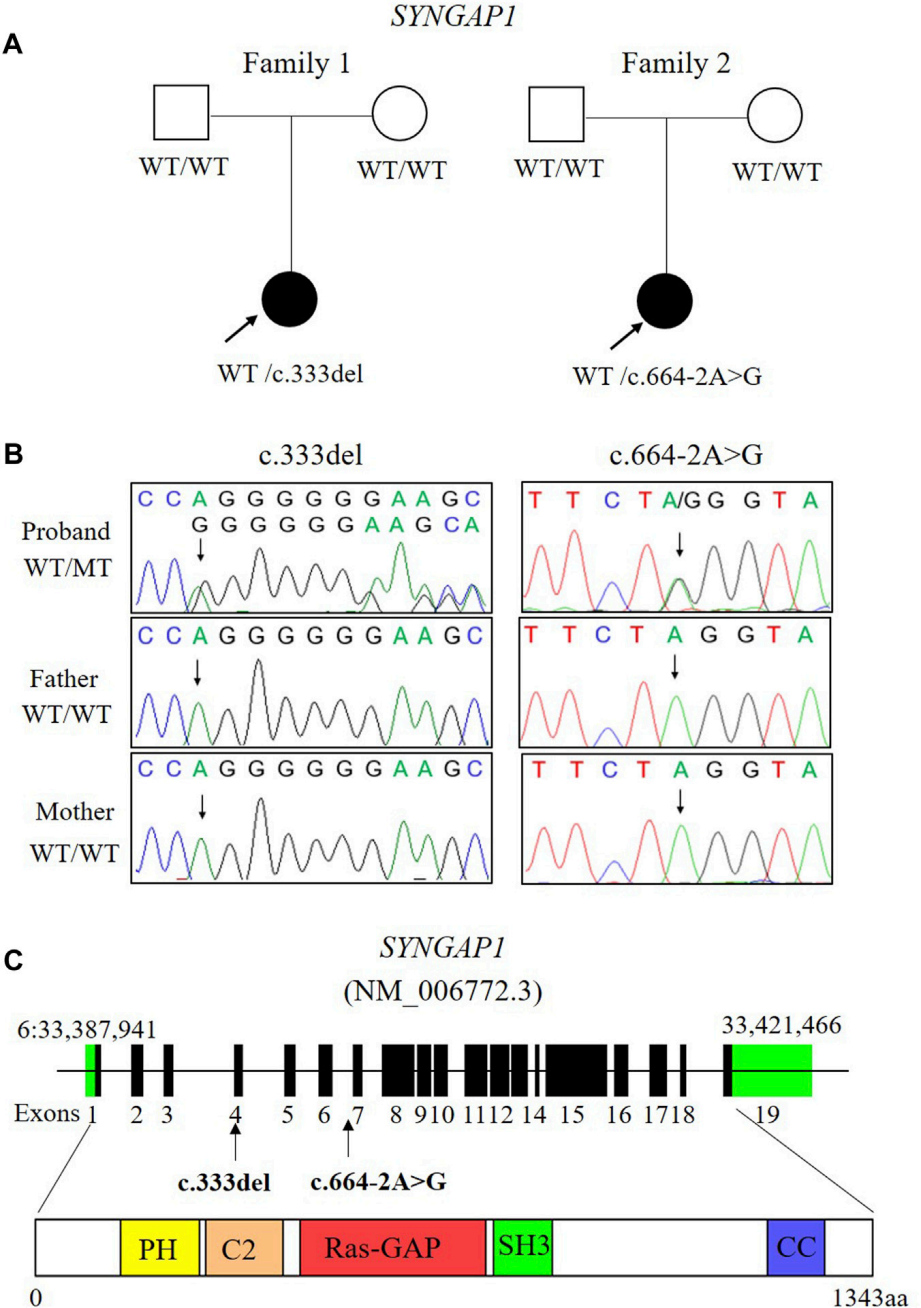
Two *de novo* variants of *SYNGAP1* were identified in two patients with ID. **(A)** Families pedigree and genotype are shown. The probands with ID underwent WES. Filled symbols represent affected individuals. **(B)** Sanger sequencing chromatograms of the *SYNGAP1* variants in these families. **(C)** Localization of the *SYNGAP1:* c. 333del and c.664-2A>G variant found in the study. The amino acid (aa) positions are referenced to RefSeq number NM_006772.3 (isoform-1: 1343 aa). Various predicted SYNGAP1 domains are showed: PH, pleckstrin homology domain (amino acid positions 150–251), C2 domain (amino acid positions 263–362), Ras-GAP (amino acid positions 392–729), SH3 (amino acid positions 785–815), coiled coil (CC; amino acid positions 1189–1262).

Two *de novo* heterozygous pathogenic variants in *SYNGAP1*, NM_006772.3: c. 333del and c.664-2A>G, were identified in two ID patients separately, as detailed in Table 1. The c.333del variant has been reported previously as a *de novo* finding in a child with ID (Carvill et al., 2013). The c.333del variant is predicted to cause loss of normal protein function either through protein truncation or nonsense-mediated mRNA decay. Conversely, the c.664-2A>G variant was a novel *de novo* intronic variant identified in a child with ID, which has not been reported in the literature and databases (ClinVar, DVD, PubMed, HGMD, etc.) (Figure 1).

**TABLE 1:** Variations of *SYNGAP1* genes identified in two ID patients and their clinical characteristics.

Patient	Age (m)	IQ	Language delay	Motor delay	Age of walking (m)	Current speech ability	Feeding difficulty	ASD	Seizures	Gait	EEG	MRI	Karyotype	Mutation type	Source of mutation	Nuclotide change	Amino acid change	ACMG	Pathogenicity
Patient 1	67	45	Yes	Yes	16	Two–three words	No	No	No	Unsteady gait	Normal	Normal	46,XX	Frameshift	*De novo*	c.333del	p.Lys114SerfsX20	PVS1+PS1+PS2+PP5	Pathogenic
Patient 2	84	55	Yes	Yes	36	Simple sentences	No	No	No	Wide-based gait	Normal	Normal	46,XX	Splicing	*De novo*	c.664-2A>G	-	PVS1+PS2+PM2	Pathogenic

Note: −, absent; m, month(s); IQ, intelligence quotient; ASD, autism spectrum disorder; EEG, electroencephalogram; MRI, magnetic resonance imaging.

### Clinical characteristics of the NSID affected individual

Both patients in their respective families, carrying *de novo* variants in *SYNGAP1*, exhibit delays in intellectual and motor developmental. These two patients had impaired speaking ability and were speech disabled, along with symptoms such as hypotonia, muscle flaccidity, and a wide-based/unsteady gait. Notably, they didn’t exhibit feeding difficulty, autism spectrum disorder (ASD), epilepsy, or microcephaly. We conducted a comprehensive assessment for ASD on these two patients, which included psychological tests, clinical examinations, and consideration of their family medical history. We utilized standardized assessment tools such as the Autism Diagnostic Observation Schedule (ADOS) for ASD evaluation and found no symptoms of ASD in these patients. Additionally, we conducted initial psychological tests on both patients to assess cognitive functioning and identify any coexisting conditions, and the results indicate that both of these patients don't exhibit symptoms of autism. Both patients had normal electroencephalograms (EEGs) and normal karyotypes (Table 1). There is no family history related to developmental disorders. The two patients are girls and are the only children in their respective families. One patient is 5 years old and the other is 7 years old, and their parents are healthy. They have experienced an overall delay in developmental milestones. For example, the patient which carrying the c.664-2A>G variant was independently sitting and walking later than children of the same age. She didn't achieve independent walking until the age of 3 years, and her gait was extensive and unstable. At the age of 7, her intelligence quotient (IQ) was measured at 55 on the Tanaka-Binet IQ Scale V. Furthermore, she exhibits delayed language development and only uses short and simple sentences with limited vocabulary.

### Expression of *SYNGAP1* mRNA in transfected cells with recombinant plasmids

**FIGURE 2:**
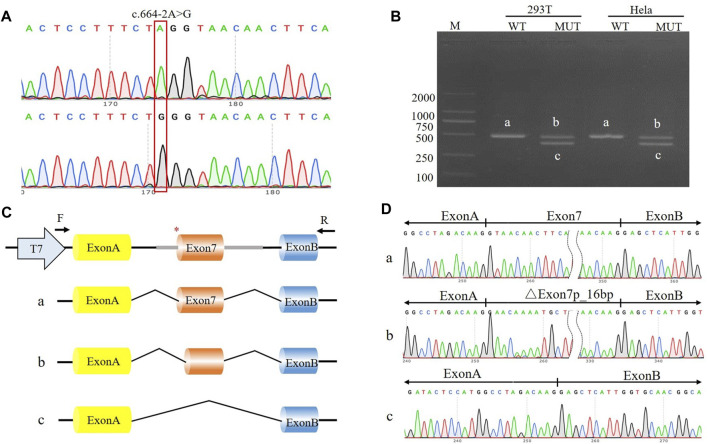
The effect of the c.664-2A>G variant on splicing was assessed through a minigene assay. **(A)** Construction of the pcMINI-*SYNGAP1*-WT/MUT vector, which contain exon 7 and flanking intronic sequences of WT or mutant type (c.664-2A>G) of the *SYNGAP1* gene. **(B)** Minigene assay performed in 293T and Hela cells transfected with the pcMINI-*SYNGAP1*-WT/MUT vector. The PCR products were isolated by gel electrophoresis. The *SYNGAP1* splicing products of wild-type (band a) and variant type (band b and c) are shown. **(C, D)** Schematic diagram of minigene construction and sanger sequencing of PCR products.

**FIGURE 3:**
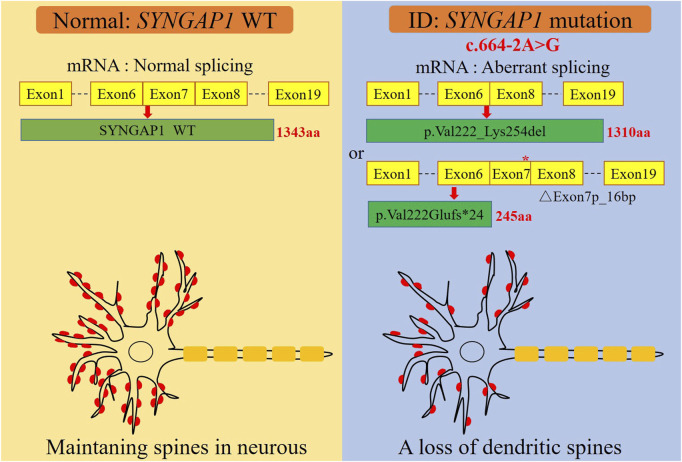
A graphical summary of the mechanism of dendritic spine loss caused by the *SYNGAP1* c.664-2A>G variant. The *SYNGAP1* c.664-2A>G variant causing aberrant splicing and dendritic spine loss.

The c.333del variant has been reported previously as a *de novo* finding, so we did not perform functional experiments on it. We performed *in vitro* experiments on the novel *de novo* splicing variant in *SYNGAP* gene. To investigate the influence of the c.664-2A>G variant on splicing, we conducted a minigene splicing assay. The pcMINI-*SYNGAP1*-WT and pcMINI-*SYNGAP1*-MUT (c.664-2A>G) plasmids were transiently transfected into 293T and HeLa cells (Figure 2A). The total RNA was extracted and reverse-transcribed into cDNA after transfection over 48 h. The cDNA was amplified by PCR and analyzed by agarose gel electrophoresis. Agarose gel electrophoresis showed that the mutant-type (MUT) had two bands, all bands were smaller than the WT band, and their migrations were relatively faster (Figure 2B). DNA sequencing results showed that the WT minigene (pcMINI-*SYNGAP1*-WT) transcribed normal mRNA composed of exon 7 (Figure 2C, band a), while the c.664-2A>G mutant minigene caused abnormal splicing, resulting in exon 7 skipping (Figures 2C, D, band b) and a 16 bp deletion within exon 7 (Figures 2C, D, band c), which reveals that this variant may be a crucial mechanism for the pathogenesis of ID. Exon 7 skipping could result in the loss of 33 amino acids (c.664_762del p.Val222_Lys254del), and the deletion of 16bp in exon 7 could result in a frameshift of amino acids at position 222 and a premature stop codon (c.664_679del p.Val222Glufs*24). The aberrant splicing is predicted to abolish the pleckstrin homology (PH, pos. 150–251aa) and C-terminal domains, suggesting that it would prevent the SYNGAP1 protein from performing its normal functions. *SYNGAP1* c.664-2A>G may damage cognitive and social adaptation development by impairing maturation of dendritic spine synapses in neurons (Figure 3).

## Discussion

In this study, we identified two *de novo* heterozygous pathogenic variants in *SYNGAP1* among 113 patients with ID. The c.333del variant has been previously reported as a *de novo* finding in a child with ID (Carvill et al., 2013), while the other splicing variant c.664-2A>G has not been reported in any literature or databases. The c.333del variant is predicted to cause loss of normal protein function either through protein truncation or nonsense-mediated mRNA decay. The phenotypes of the ID patient, which carrying the c.664-2A>G variant, are similar to the previously published truncating variant in *SYNGAP1*. Although the intronic variants may have more deleterious effects than the exonic variants, they are underexplored (Kallel-Bouattour et al., 2017). To further evaluate the deleterious effects of the intronic variant c.664-2A>G, we conducted a minigene assay to investigate its impact on mRNA splicing. The minigene experiment results showed that the intronic variant c.664-2A>G causes aberrant splicing of *SYNGAP1.* The c.664-2A>G variant would result in exon 7 skipping and partial exon 7 deletion, which would abolish critical functional domains and impair the function of the SYNGAP1 protein.

There is a potential acceptor site located 16 bp upstream of exon 7 in *SYNGAP1*. After c.664-2A>G variant, this site is activated for splicing. The c.664-2A>G variant disrupts the original acceptor site, potentially leading to the recognition of the alternative splicing site that causes a 16 bp deletion on upstream of exon 7. Alternatively, the disruption of the original acceptor site might result in direct skipping of exon 7 during splicing, resulting in the overall deletion of exon 7, much like how a gene in a database might have multiple normal transcripts.

*SYNGAP1* is an important gene that is necessary for neuronal development, and its dysfunction is associated with ID (Jeyabalan and Clement, 2016). *SYNGAP1* is located on human chromosome 6, contains 19 exons, and generates approximately a 6 kb transcript, which encodes a brain-specific synaptic Ras GTP-ase activating protein. The impairment of SYNGAP1 function may make patients with ID susceptible to seizures by increasing the recruitment of AMPA receptors at postsynaptic glutamatergic synapses, which leads to increased transmission of excitatory synapses (Hamdan et al., 2009). In large-scale studies, almost all *SYNGAP1* variants associated with NSID, ASD, and epilepsy are loss-of-function and lead to SYNGAP1 haploinsufficiency, resulting in intellectual developmental disorder (Hamdan et al., 2011; Berryer et al., 2013; Carvill et al., 2013; Fieremans et al., 2016).In our study, as well as previous observations, suggest that the *SYNGAP1* c.664-2A>G variant would cause ID mainly through a mechanism of haploinsufficiency.

NSID poses a challenge for clinicians because of the absence of specific clinical features to guide them toward an etiological diagnosis. The identification of novel variants in known pathogenic genes or novel ID genes suggests that molecular diagnostic approaches are becoming increasingly significant in unraveling the underlying causes of this condition. In the present study, the two patients presented with comprehensive developmental delays, particularly motor milestones and language development, and exhibited behavioral disorders. We identified pathogenic variants in *SYNGAP1* in both of these patients by WES. Based on clinical and genetic features, the patients were diagnosed with intellectual developmental disorder. *De novo SYNGAP1* variants were initially reported to cause ID, accounting for approximately 0.62% of all the patients in the Deciphering Developmental Disorders (DDD) study (Hamdan et al., 2011; Wright et al., 2015). Six patients with *SYNGAP1* variants exhibited moderate to-severe ID due to severe language impairment (Hamdan et al., 2011). Studies involving rodent models with the deletion of the *SYNGAP1* allele showed abnormal formation and maturation of dendritic spines in neurons, altered excitatory-inhibitory (E/I) balance, and changed the critical period of development, suggesting that heterozygous variants also have the potential to disrupt brain function in humans and lead to ID through the mechanism of haploinsufficiency (Rumbaugh et al., 2006; Guo et al., 2009; Muhia et al., 2010).

In conclusion, we identified two *de novo* pathogenic heterozygous variants in *SYNGAP1,* c. 333del and c.664-2A>G, among which the c.664-2A>G variant was a novel *de novo* pathogenic variant. Based on previous findings from others and our research, nonsense variants in *SYNGAP1* remain the most common variant type leading to ID. This study further enriched the variant landscape of *SYNGAP1* in ID and provided a basis for the clinical diagnosis and genetic counseling of ID.

## Data Availability

The original contributions presented in the study are publicly available. This data can be found here: https://ngdc.cncb.ac.cn/search/?dbId=hra&q=HRA005421.
